# Pneumoparotid causing subcutaneous emphysema secondary to chronic habitual Valsalva behaviours in a 13-year-old girl

**DOI:** 10.1093/bjrcr/uaaf048

**Published:** 2025-09-18

**Authors:** Jessica Carter, Jody Maclachlan, Peter Wylie, Shilen Patel, Sherif Hanna

**Affiliations:** Department of Radiology, Royal Free NHS Foundation Hospital Trust, London, NW3 2QG, United Kingdom; Department of Radiology, Royal Free NHS Foundation Hospital Trust, London, NW3 2QG, United Kingdom; Department of Radiology, Royal Free NHS Foundation Hospital Trust, London, NW3 2QG, United Kingdom; Department of Maxillofacial Surgery, Royal Free NHS Foundation Hospital Trust, London, NW3 2QG, United Kingdom; Department of Radiology, Royal Free NHS Foundation Hospital Trust, London, NW3 2QG, United Kingdom

**Keywords:** Pneumoparotid, parotid gland, subcutaneous emphysema, pediatrics, ENT

## Abstract

Pneumoparotid is an unusual condition involving retrograde insufflation of air via the mouth into the parotid gland. In rare circumstances it can be complicated by subcutaneous emphysema. We describe a highly unusual case of rapid-onset facial swelling and limited eye-opening in an otherwise clinically well 13-year-old female. Computed tomography imaging showed pneumoparotid and extensive surgical emphysema involving the cervicofacial soft tissues and mediastinum. A detailed history revealed habitual Valsalva-related behaviours, including ear-popping and blowing bubbles in drinks. She was diagnosed with subcutaneous emphysema secondary to pneumoparotid, and her symptoms resolved with conservative management.

## Introduction

Pneumoparotid is a rare phenomenon caused by the retrograde insufflation of air via the mouth through the parotid (Stensen’s) duct, resulting in gas accumulation within the parotid gland. This is considered a predominantly non-inflammatory condition; however the reflux of saliva into the gland may lead to concomitant infection and parotitis.^1^

The most common cause of parotid swelling in children is infective parotitis secondary to the mumps virus.^2^ The second most common cause is juvenile recurrent parotitis, an inflammatory condition with unknown aetiology.^2^ Other known causes of parotitis include sialoliths, Sjögren syndrome, lymphoma, and HIV.^2^^,^^3^

### Causes

Pneumoparotid has various causes, including iatrogenic factors such as anaesthetic induction procedures, continuous positive airway pressure (CPAP) devices, and dental treatments involving air insufflation, all of which increase intraoral pressure via external devices.^3–5^ Occupational factors result from playing wind instruments such as trumpets,^4^ or blowing up balloons.^3^ Behavioural causes may involve Valsalva manoeuvres such as habitual puffing out of cheeks and are more commonly seen in children.^5^^,^^4^ Factitious causes have notably included self-induced pneumoparotid in army officers in 1918 to simulate mumps to avoid duty, referred to as ‘factitious mumps’.^4^ Respiratory conditions, such as cystic fibrosis, can also lead to pneumoparotid due to recurrent cough or cough suppression.^3–5^ Psychiatric conditions with behaviours of highly repetitive puffing of cheeks or other Valsalva-like behaviours. The remaining cases are idiopathic with no clear cause.^4^^,^^5^

There may be inherent anatomical abnormalities which predispose to pneumoparotid where the mucosal protection sealing the entrance to the gland is affected. These include damage or dilation of the salivary duct and duct ostium, an abnormally patulous parotid orifice, and weakness of the buccinator or hypertrophy of the masseter muscles.^1^^,^^3^^,^^5^

### Clinical characteristics and complications

A recent review of the clinical findings in 170 cases of pneumoparotid found that the most common symptom of pneumoparotid is swelling, followed by pain.^3^ Under half of the patients had palpable crepitus, and the most notable clinical examination finding was visible frothy saliva at the opening of Stensen’s duct.^3^ Laterality was almost equally distributed, with no significant difference in the proportion of bilateral, left-sided, or right-sided presentations of pneumoparotid.^3^

A rare complication of pneumoparotid is rupture of gas through the gland acini, leading to the extension of gas into the soft tissues, causing surgical emphysema. This complication is typically limited to the cervicofacial soft tissues, but in even rarer cases gas can track into the mediastinum,^4^ as is described in this case.

### Management

Immediate management of pneumoparotid involves conservative measures, including gland massage, warm compresses, hydration, and sialogogues. Symptoms typically resolve within 72 hours.^5^ Prophylactic antibiotics may be used to prevent infection from oral bacteria. Definitive management depends on the patient’s specific aetiology and removing causative factors. Surgical interventions, including duct ligation, may be reserved for chronic or recurrent cases.^6^ Less invasive options such as sialendoscopy and steroid irrigation may be considered in select patients.^7^

## Case report

### Clinical presentation

A 13-year-old girl presented to the emergency department with a sudden onset of left-sided painful facial swelling over a few hours. This was on a background of 2 weeks’ existing bilateral parotid swelling with a neck ultrasound, also 2 weeks prior, demonstrating a right-sided parotitis and a normal left parotid gland. Five days prior to the current presentation, she was discharged after a brief period of observation and antibiotics. At that time her serum amylase was markedly raised at 1591 Units/L (Normal range 28-100 Units/L).

She denied fevers, headache, and cold symptoms and had seen her dentist within the last year for an examination and dental radiographs, which were normal. She had no allergies, was up to date with her immunisations and was otherwise in good health with a past history of mumps at 1 year old. She has no history of psychiatric illness.

On assessment her vital signs were within the normal range. On examination she had marked swelling over the left jaw and parotid gland, extending down the left side of the neck and superiorly over the temple and eye, with limited left eye opening to a few millimetres. There were no overlying skin changes. Mouth opening was limited by pain to two finger widths, but oral examination was normal with no evidence of discharge from Stensen’s duct. Her respiratory and abdominal examinations were also normal. Inflammatory markers were not elevated, however, serum amylase was mildly raised at 106 Units/L (Normal range 28-100 Units/L).

This attendance was her seventh presentation to the accident and emergency department over 2.5 years with intermittent relapsing and remitting facial swelling and pain, localised over the parotid glands. The three most recent prior presentations involved the left side of the jaw and face; however, instances before these involved the right side. She was constitutionally well during all her prior episodes, and she was diagnosed with parotitis, for which she was prescribed oral antibiotics. She had various outpatient follow-up appointments with ENT and underwent a number of ultrasounds showing either normal parotid glands or evidence of unilateral parotitis.

### Imaging

**Figure 1.**
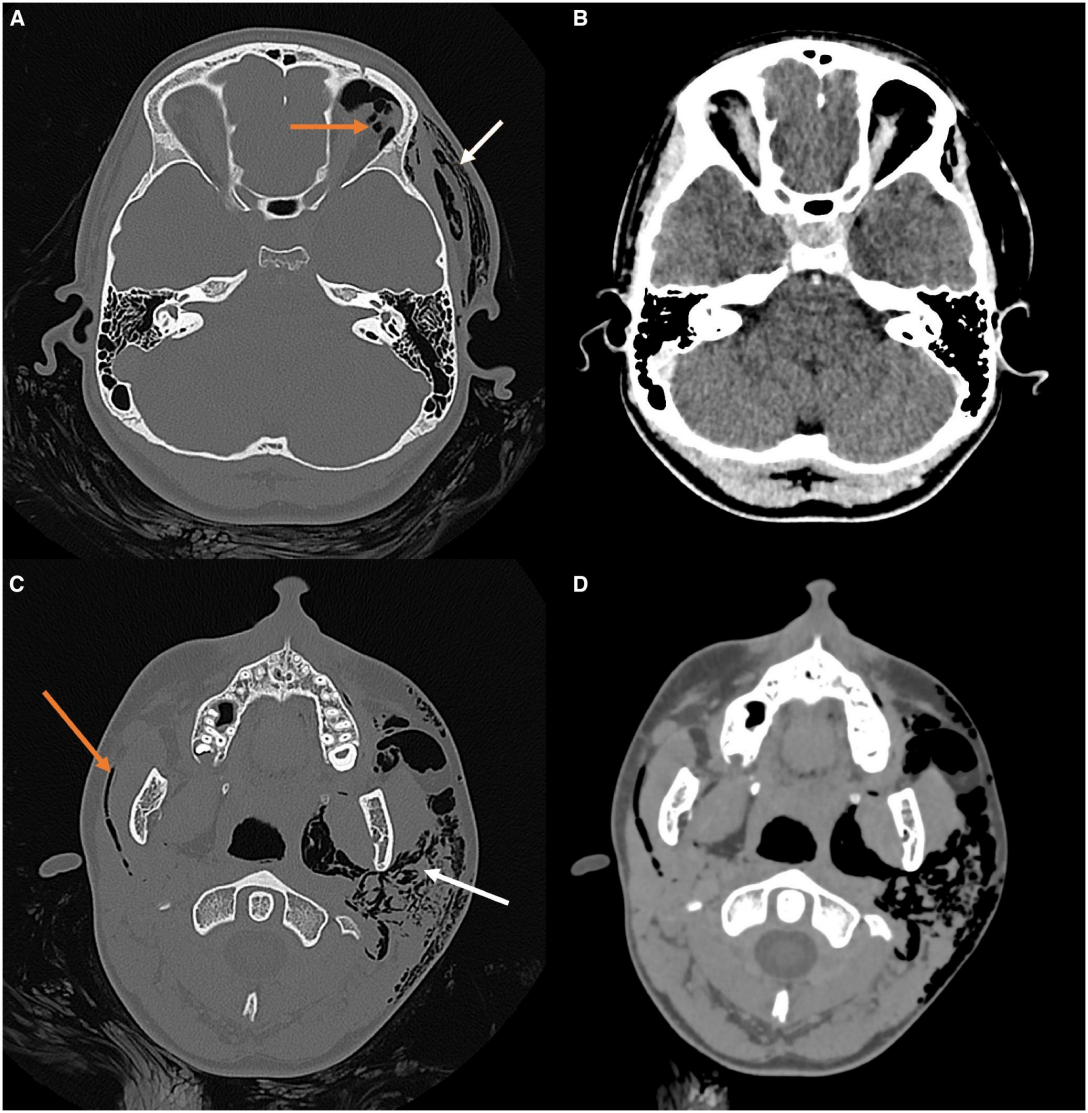
Non-contrast CT orbits. (A) Subcutaneous gas seen in the soft tissues of the left scalp (white arrow) and left periorbital and extraconal spaces (orange arrow). (B) No other brain or soft tissue abnormality evident on brain windows of the same slice. (C) Extensive gas disseminated throughout the left parotid gland (white arrow), dissecting into the adjacent subcutaneous tissues. Notably, there is gas delineating the right Stensen’s duct (orange arrow). (D) On soft tissue windows of the same slice, the right parotid gland was normal.

Due to the painful and pronounced facial swelling, CT imaging of the orbits was performed. This demonstrated extensive, predominantly left-sided subcutaneous emphysema involving the parapharyngeal spaces, which tracked along the left face, orbit, extraconal space, and scalp (Figure 1). Gas was predominantly centred within and around the **left** parotid gland; however, a smaller volume of gas was noted outlining the **right** Stensen’s duct adjacent to an otherwise normal-looking right parotid gland (Figure 1). These findings were consistent with pneumoparotid, with a differential that included gas-forming infection. In light of these dramatic CT findings and the continued clinical progression of her facial swelling while in the department, the patient was started on intravenous antibiotics.

**Figure 2.**
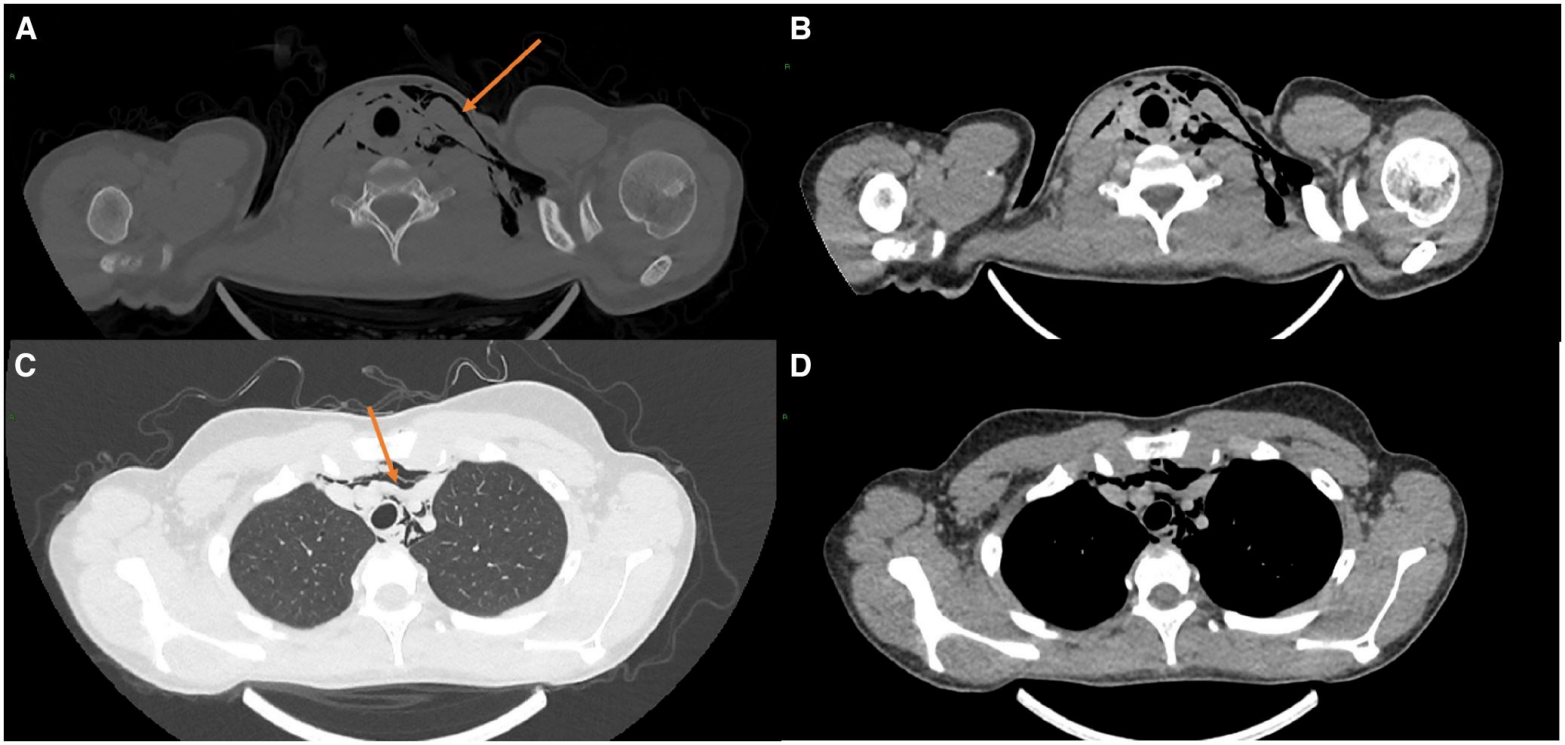
Non-contrast CT thorax: (A) Surgical emphysema predominantly noted in the left neck on bone windows (arrow). (B) Otherwise normal tissue appearances seen on soft tissue windows of the same slice. (C) Mediastinal emphysema evident more inferiorly (arrow) with normal appearance of the lung apices on lung windows. (D) Soft tissue windows of the same slice with otherwise normal soft tissue appearances.

CT imaging of the thorax was subsequently performed to assess the extent and investigate other causes of the surgical emphysema. This demonstrated further extension of the subcutaneous emphysema inferiorly into the neck soft tissues and mediastinum, with a small volume of gas abutting the oesophagus and carina (Figure 2). The lungs were normal, with no pneumothorax, and no CT evidence of oesophageal abnormality or free fluid in the mediastinum was seen.

### Outcome and follow-up

The patient’s facial swelling improved spontaneously, and she was discharged after 5 days on oral antibiotics. On outpatient follow-up, after further specific questioning about other Valsalva behaviours, it transpired that she enjoyed blowing bubbles into water and would regularly pressure-equalise her ears. This additional information in context with her normal bloods and observations led to a confident diagnosis of subcutaneous emphysema secondary to pneumoparotid due to habitual Valsalva activities. As such, she was counselled to discontinue all pressure-related activities. The patient had made a good recovery with no further episodes of parotitis when reviewed in an outpatient clinic 10 months later.

## Discussion

### Diagnostic difficulty

The diagnosis of pneumoparotid in this patient was challenging, as historically her symptoms would seemingly remit after courses of antibiotics, which was more suggestive of parotitis. Indeed pneumoparotid can coexist with parotitis due to the retrograde introduction of bacteria into the gland which confounds the diagnosis of pneumoparotid. Additionally, this patient did not exhibit the classical ‘foamy secretions’ on examination of the mouth and on initial information gathering she denied the typical precipitants for pneumoparotid. It was only on very specific questioning that the Valsalva behaviours were elucidated, highlighting the importance of careful history-taking.

The patient had normal inflammatory markers, and the only abnormal blood result was a raised serum amylase. Of note, elevated amylase has been described in other similar case reports, including pneumoparotid and surgical emphysema in a 14-year-old boy who developed acute onset facial swelling after starting to play the tuba.^8^ Damage to the parotid gland following pneumoparotid is assumed to explain this finding. Interestingly our patient had raised serum amylase with parotid swelling 2 years prior to this attendance, and at that time she also tested negative for active mumps infection. It is presumed that her multiple previous presentations were secondary to pneumoparotid. We also note that she has a history of mumps in infancy. The significance of this is uncertain as there is no described association in the limited pneumoparotid cases reported.

### The role of imaging

There is no current imaging standard for the diagnosis of pneumoparotid. CT is the most sensitive modality to detect and delineate gas in the parotid duct and surrounding tissues. A notable case example in the literature describes using CT baseline images and subsequent repeat acquisition with puffing out of cheeks to demonstrate the reflux of gas into the glands.^5^ Routine CT use is limited in the paediatric population due to radiation concerns. Other imaging modalities include sialography, a standard investigation for sialectasia, ductal stones, and strictures. Gas can be detected on a plain radiograph,^5^^,^^9^ and gas bubbles are easily seen directly on sialendoscopy, which is useful to detect other parotid duct pathologies.^10^

Ultrasound can demonstrate gas in the parotid gland, as the relatively low acoustic impedance of air bubbles compared to the surrounding tissues appears as hyperechoic reflexes. A recent study characterising the ultrasound findings in 14 cases of confirmed pneumoparotid describes in every case observing “*multiple small, flattened, mobile, hyperechoic reflexes within the parenchyma*” with posterior acoustic shadowing, albeit to varying degrees.^10^ Gland massage could also demonstrate shifting of the shadows. While ultrasound is quick and safe, it requires there to be gas present within the gland at the time of the examination and depends upon user skill.

**Figure 3.**
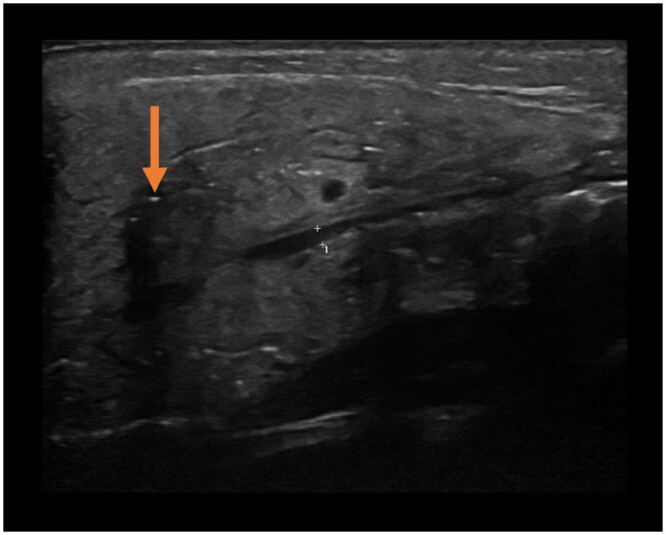
Ultrasound image of the right parotid gland acquired prior to CT. This demonstrates multiple tiny echogenic foci (arrow) with associated posterior acoustic shadowing, presumed to represent intra-gland gas.

Comparing our patient’s previous static ultrasound images, tiny echogenic foci related to the parotid gland prior to treatment may be attributed to intra-gland air (Figure 3). However, it would be difficult to confidently make this rare diagnosis on ultrasound alone.

## Learning points

- Pneumoparotid is a rare phenomenon that is commonly misdiagnosed as parotitis.
- Diagnosis requires careful history-taking to identify causal behaviours or occupations.
- Key clinical findings of pneumoparotid include facial pain, swelling, and frothy secretions at Stensen’s ducts.
- Rare complications include subcutaneous emphysema of the adjacent cervicofacial soft tissues and the mediastinum.
- There is no imaging standard to diagnose pneumoparotid, however gas can be detected on ultrasound where user expertise allows.
